# Metabolic Implications of Oxidative Stress and Inflammatory Process in SARS-CoV-2 Pathogenesis: Therapeutic Potential of Natural Antioxidants

**DOI:** 10.3389/fcimb.2021.654813

**Published:** 2021-05-26

**Authors:** Gilead Ebiegberi Forcados, Aliyu Muhammad, Olusola Olalekan Oladipo, Sunday Makama, Clement Adebajo Meseko

**Affiliations:** ^1^ Biochemistry Division, National Veterinary Research Institute (NVRI), Vom, Nigeria; ^2^ Department of Biochemistry, Ahmadu Bello University, Zaria, Nigeria; ^3^ Regional Laboratory for Avian Influenza and Other Transboundary Animal Diseases, National Veterinary Research Institute, Vom, Nigeria

**Keywords:** COVID-19, oxidative stress, inflammation, therapeutics, antioxidants

## Abstract

COVID-19 is a zoonotic disease with devastating economic and public health impacts globally. Being a novel disease, current research is focused on a clearer understanding of the mechanisms involved in its pathogenesis and viable therapeutic strategies. Oxidative stress and inflammation are intertwined processes that play roles in disease progression and response to therapy *via* interference with multiple signaling pathways. The redox status of a host cell is an important factor in viral entry due to the unique conditions required for the conformational changes that ensure the binding and entry of a virus into the host cell. Upon entry into the airways, viral replication occurs and the innate immune system responds by activating macrophage and dendritic cells which contribute to inflammation. This review examines available literature and proposes mechanisms by which oxidative stress and inflammation could contribute to COVID-19 pathogenesis. Further, certain antioxidants currently undergoing some form of trial in COVID-19 patients and the corresponding required research gaps are highlighted to show how targeting oxidative stress and inflammation could ameliorate COVID-19 severity.

## Introduction

The coronavirus disease 2019 (COVID-19) is a multi-organ disease caused by severe acute respiratory syndrome coronavirus-2 (SARS-CoV-2). In March 2020, the World Health Organization (WHO) declared the coronavirus disease (COVID-19) a pandemic with about 571,678 confirmed cases and 26,494 deaths declared globally at that time (Chow et al., 2020). Many countries in the world are currently experiencing a second wave of the disease with several mutations of the virus, high rate of transmission and consequent high mortality rates. Droplet transmission is reportedly the main transmission route of the virus, but there are also suggestions that the virus could be airborne (Basu-Ray and Soos, 2020). The major clinical manifestations in patients include fever, dry cough and headache, while loss of smell and taste has been observed in some patients (Aggarwal et al., 2020).

SARS-CoV-2 is enveloped virus with a single positive-stranded RNA genome of approximately 26-32 kb (Li L. et al., 2020). The coronaviruses are a large group of viruses comprising of four subfamilies; alpha, beta, gamma and delta. However, it is the beta-coronaviruses to which SARS-CoV-2 belongs that cause the most severe morbidity and fatality (Suhail et al., 2020). It has been shown that four structural proteins are encoded in all coronaviruses: the spike protein, nucleocapsid protein, membrane protein and the envelope protein. Among these structural proteins, the spike protein is the largest and is required for viral entry into host cells (Casalino et al., 2020). SARS-CoV-2 entry into a host cell involves a number of conformational changes requiring binding of a virus particle to angiotensin-converting enzyme (ACE-2) receptor on the cell surface and fusion of the viral envelope to the cell membrane (Guo et al., 2020).

A combination of factors comes into play to make viral entry into a host cell and subsequent replication in the host possible (Suhail et al., 2020). A number of conformational changes are required for the successful binding and entry of an encapsulated virus into a host cell (Suhail et al., 2020). The redox status of a host cell which is determined by the oxidant/antioxidant balance contributes to the stability of the proteins and interactions on the host cell surface (Fenouillet et al., 2007). Even after successful entry of the virus into the host, the innate immune system recognizes the foreign genome and responds by activating macrophage and dendritic cells which use reactive oxygen/nitrogen radicals and cytokines that can contribute to inflammation and further exacerbate the host system response in favor of COVID-19 progression (Bohn et al., 2020). Hence, the implications of oxidative stress versus ‘cytokine storm’-induced inflammation vis-à-vis the potential of natural antioxidants in ameliorating the ultimate fatal outcomes cannot be overemphasized. Consequently, this review provides mechanistic insight into the role of oxidative stress and inflammation in COVID-19 pathogenesis and proposes mechanisms by which targeting these processes by natural antioxidants could ameliorate pathologies associated with the condition.

## Oxidative Stress and SARS-CoV-2 Pathogenesis

Oxidative stress is a physiological state in which systemic levels of reactive oxygen species (ROS) overwhelms the cellular antioxidant buffering capacity, eventually resulting in damage to cellular macromolecules (Alkadi, 2018). The production of ROS and free radicals occur during normal cellular metabolism. Under normal physiological conditions, ROS play beneficial roles in important signaling pathways required for essential cellular functions (Alpay et al., 2015). The mitochondria generate ROS during coupling reactions that form ATP when molecular oxygen is reduced to superoxide anion $\left(O_{2}^{-}\right)$ in the electron transport chain. The interaction of superoxide anion with transition metals like Fe^2+^ can result in the production of other radicals like hydrogen peroxide (H_2_O_2_), hydroxyl radical (OH^•^), and organic peroxides. The mitochondrial respiratory chain also produces nitric oxide (NO), which can generate other reactive nitrogen species (Valko et al., 2007).

Reactive oxygen species play a number of important roles in normal cell physiology. In endothelial cells, angiotensin II stimulates $O_{2}^{-}$ generation through activation of NAD(P)H oxidase (Nguyen Dinh et al., 2013). Downstream, $O_{2}^{-}$ activates the Raf-1 mitogen activated protein kinase (MAPK) resulting in activation of proteins that control cell proliferation (Harijith et al., 2014). In the central nervous system, $O_{2}^{-}$ contributes to increased vasopressin secretion which controls blood pressure (Gonzalez et al., 2020). The role of ROS in vasodilation is seen in NO which functions as a signaling molecule when produced at low concentrations in vascular endothelial cells by the constitutive isoform of nitric oxide synthase (Pisoschi and Pop, 2015). Macrophages also utilize nitric oxide synthase to generate NO that is used for killing invading pathogens. In smooth muscle tissue, NO activates relaxation of corporal cavernosal resulting in increased blood flow that sustains penile function required for reproduction (Castela and Costa, 2016). The cytokine, tumor necrosis factor-α (TNF-α) also induces generation of mitochondrial ROS implicated in apoptotic cell death (Doss et al., 2014). These examples point to the beneficial roles of ROS in normal cell physiology.

On the other hand, the deleterious effects of reactive species can be seen in the oxidation of cellular macromolecules like lipids, proteins and carbohydrates resulting in alterations in their functions. The hydroxyl radical, which is highly reactive with a short half-life can react with DNA bases to form adducts that can alter transcription resulting in altered protein function (Thimmulappa et al., 2019). Peroxyl radicals also contribute to DNA cleavage and protein backbone modification by synergistically enhancing DNA damage by superoxide anion (Liou and Storz, 2010). When such oxidative damage to DNA occurs, molecules like 8 -hydroxy deoxyguanosine (8-OHdG) are generated which increase the risk of mutagenesis (Matsui et al., 2000). The compound 8-OHdG has been implicated in the initiation and promotion stages of carcinogenesis as it is reportedly increased eight to seventeen folds in breast tumors when compared to non-malignant breast tissue (Matsui et al., 2000). Also, the compound can induce GC → TA transversion mutations particularly during DNA replication, with a potential for mutagenicity, if the oxidative lesions are not repaired, resulting in cancer initiation. DNA adducts formation in the coding region of tumor suppressor proteins like TP53, can cause altered function, resulting in cancer promotion, progression and metastasis (Wei et al., 2012). Although there is inadequate information on DNA adduct levels in COVID-19 patients, it is possible that elevated levels of oxidative stress reported in COVID-19 patients could cause DNA oxidation and other downstream effects (Cecchini and Cecchini, 2020).

Oxidation of membrane lipids results in the formation of malondialdehyde (MDA) and 4-hydroxynonenal that affect the compactness and integrity of cell membranes (Niki, 2014). Oxidation of membrane lipids by ROS causes changes to the inner mitochondrial membrane integrity resulting in opening of the mitochondrial permeability transition (MPT) pore, loss of the mitochondrial transmembrane potential, release of cytochrome *c* and eventually death of the cell (Yadav et al., 2015). Lipids are very susceptible to peroxidation by oxidants, especially the polyunsaturated fatty acids that contain a number of double bonds. Since lipids constitute the membrane bilayer, attack on lipids by reactive intermediates leads to a cascade of reactions called lipid peroxidation (Ray et al., 2000). This alters the permeability and function of the membrane as well as the immunity of the cell and animal as a whole. Since lipid peroxidation is a primary event during cell injury, a number of disease conditions including diabetes, cancer, atherosclerosis, ischemia-reperfusion, heart failure, Alzheimer’s disease, rheumatic arthritis and some immunological diseases are associated with increased formation of lipid peroxides and aldehydes (Sadati Zarrini et al., 2016). A cross-sectional study of COVID-19 patients in a hospital reported elevated levels of malondialdehyde, which was attributed to oxidative stress (Muhammad et al., 2021).

Oxidation of proteins, especially those rich in cysteine-residues results in modifications that cause protein aggregation and altered function due to negative metabolic impacts on protein’s three dimensional structure (Höhn et al., 2014). The hydroxyl radical has been shown to cause oxidative attack of polypeptide backbone through abstraction of hydrogen from amino acid residues resulting in the formation of carbon-centered radicals which can rapidly react with molecular oxygen to form alkoxyl radicals that can cause protein cross linkage and fragmentation (Höhn et al., 2014). The alkoxyl radicals formed from protein oxidation by reactive oxygen species can further perpetrate the oxidation of other cellular macromolecules. Aging and aging related disorders like Alzheimer’s disease, respiratory distress syndrome, cataract formation and muscular dystrophy are significantly associated with accumulation of oxidized proteins resulting in decreased activities of enzymes required for important physiological functions (Garcia-Garcia et al., 2012). Literature search did not provide enough information on levels of oxidized proteins in COVID-19 patients, but it is possible that increased ROS levels observed in COVID-19 patients could cause oxidation of proteins due to increased apoptosis, necrotic cell debris and pulmonary interstitial fibrosis observed during analysis of postmortem lung sections of fatal COVID-19 patients (Donia and Bokhari, 2021).

Cysteine and methionine are particularly sensitive to oxidation by ROS resulting in the formation of disulfides (Bin et al., 2017). A prominent effect of this is seen in receptors that are rich in cysteine residues like the mitogen activated protein kinases, insulin and insulin-like growth factor receptors and ion channels which undergo dimerization and autoactivation in the absence of signaling molecules, resulting in dysregulated function (Bin et al., 2017). Reactive oxygen species have been reported to play roles in the activation of nuclear factor kappa beta (NF-κB), a transcription factor that regulates cytokine production, inflammation and innate immunity (Karin and Greten, 2005). Under normal physiological conditions, NF-κB is sequestered in the cytoplasm by inhibitor kappa beta (IκB), an inhibitory protein of the chaperone family of proteins (Liu et al., 2017). In response to appropriate stimulus, IκB is phosphorylated by inhibitor kappa kinase, leading to the liberation of NF-κB, which translocates to the nucleus to ensure controlled response to the stimulatory signal (Liu et al., 2017). However, this tight control mechanism can be lost during oxidative stress as hydrogen peroxide and other reactive oxygen species can oxidize the cysteine residues of NF-κB resulting in altered activation of NF-κB *via* mechanisms independent of IκB phosphorylation (Sun, 2011).

Altered activation of NF-κB results in a number of downstream effects due to the role of NF-κB as a transcription factor (Sun, 2011). In addition to controlling immune and inflammatory responses, NF-κB also plays a role in cell survival by antagonizing apoptosis associated with tumor necrosis factor-α receptors mediated stimulation of Jun N-terminal kinase (JNK) (Darnay et al., 1998). The JNK pathway is one of the signaling cascades of the mitogen activated protein kinases which controls cell proliferation, cytokine production and apoptosis (Mehan et al., 2011). Upstream of JNK, the apoptosis signal regulating kinase 1 (ASK-1) activates JNK in response to stress conditions (Kanamoto et al., 2000). Cells are constantly exposed to stress, and stress such as radiation resulting in DNA damage results in activation of DNA damage repair proteins. However, when such DNA damage is irreparable, the cell is programmed for death, executed by the JNK pathway either through activation of death signaling or inhibition of cell survival signaling (Matsuzawa et al., 2002). Dysregulated JNK activity is prominently seen during oncogenic transformation, neurodegenerative disorders, ischemia induced cell death and reperfusion injury (Kanamoto et al., 2000). Considering the role of NF-κB in the mediation of cell death which is a prominent feature of COVID-19 pathogenesis, it has been suggested that targeting NF-κB could provide therapeutic effects in patients (Guisado-Vasco et al., 2020).

The physiologic role of reactive species is also evident from both innate and adaptive immune mediated reactive species generation that targets pathogen infiltration into the host system (Lam et al., 2010). During exposure to pathogens, phagocytes generate ROS *via* oxidative burst to attack pathogens (Li et al., 2021). If some pathogens escape this response, adaptive immune response is then initiated, which uses pathogen-derived antigenic peptides produced by phagocytosis and digestion that are presented to T lymphocytes (Gasteiger and Rudensky, 2014). The activated T lymphocytes then proliferate and differentiate to produce immune effector cells that are capable of mounting an efficient and antigen-specific immune response (Brownlie and Zamoyska, 2013). In COVID-19 patients, decreased T cell counts, especially of CD8+ T cells has been reported and implicated in severity of disease pathogenesis (Liu J. et al., 2020).

The limited genome size of viruses confers an advantage in utilizing host cellular environment in favor of viral replication and proliferation, because all the requirements for viral metabolism are provided for by the host metabolic machinery (Chaitanya, 2019). To facilitate replication and proliferation, viruses induce redox imbalance that enhances viral pathogenesis (Reshi et al., 2014). A number of viral proteins are reported to produce ROS and maintain the redox state of host cell in favor of viral activity mechanisms involving the manipulation of nuclear factor erythroid-related factor 2 (Nrf2) (Ivanov et al., 2011). Nrf2 is an antioxidative transcription factor which binds to the antioxidant response elements in the promoter region of gene targets like heme oxygenase-1, glutathione-s-transferase, glutathione peroxidase-1 and catalase. In human immunodeficiency virus type 1 (HIV-1), the viral gp120 protein has been implicated in induction of oxidative stress leading to activation of the Nrf2 pathway, suggesting that modulation of Nrf2 pathway could provide possible strategies for antiviral activity (Reshi et al., 2014). For hepatitis C virus, viral proteins like NS3 and NS5A have been implicated in the induction of oxidative stress in human hepatoma cells resulting in hepatocellular damage (Ivanov et al., 2011). Increased ROS production has also been observed in influenza virus induced lung injury. Virus infected human bronchial adenocarcinoma cells were found to have a lower amount of activated Nrf2 in the nucleus, suggesting viral modulation of host antioxidant response (Reshi et al., 2014). In biopsies obtained from COVID-19 patients, suppression of NRF2 antioxidant gene was observed (Olagnier et al., 2020). The authors further observed that treatment of cells with NRF2 agonists induced a strong antiviral response that limited SARS-CoV-2 replication, pointing to the potential role of Nrf2 in the management of COVID-19 (Olagnier et al., 2020).

The redox status of a host cell is an important factor in viral entry due to the unique conditions required for the conformational changes that ensure the binding and entry of an encapsulated virus into the host cell (Fenouillet et al., 2007). Basically, disulfide-thiol balance determines the redox status of a cell and can affect the pH and stability of proteins. Studies have shown that alterations to the native disulfide state especially at the surface of a target cell significantly affect cell-virus interactions in SARS-CoV-2 pathogenesis (Suhail et al., 2020). Specifically, it has been reported that increased thiol level at the SARS-CoV-2 spike protein-host cell membrane interface decreased viral binding capacity pointing to a thiol quantity needed for entry of the virus into the host cell (Suhail et al., 2020). It has been suggested that thiol and disulfide groups at the virus-host cell interface could serve as electron donors or acceptors required for the conformational changes that make viral fusion and entry into host cells possible (Suhail et al., 2020).

The activity of angiotensin-converting enzyme 2 (ACE-2) which is vital in host entry, amplification and subsequent pathogenesis of SARS-CoV-2 can also contribute to oxidative stress in the pathogenesis of the disease (Suhail et al., 2020). ACE-2 is a membrane bound receptor expressed in cells of different tissues. This protein is responsible for degrading angiotensin II which is a vasoconstrictor that can increase superoxide levels and reactive oxygen species. The entry of SARS-CoV-2 into host cells is reportedly dependent on binding of the spike protein of the virus to ACE-2 on the host cell membrane (Suhail et al., 2020). Binding of ACE-2 to the spike protein increases the cellular concentration of angiotensin II due to reduced ACE-2 degrading of angiotensin II. The increased levels of angiotensin II in such a situation increases the level of superoxide species and could contribute to oxidative stress and oxygen deprivation in the patients, resulting in cellular damage that drives COVID-19 progression requiring oxygen therapy. Further, oxidative stress can cause oxidation of the cysteine residues on proteins of both the virus and ACE-2 to form disulfides that increase the affinity of the SARS-CoV-2 for ACE-2, thereby exacerbating COVID-19 pathogenesis (Suhail et al., 2020). Unfortunately, COVID-19 being a multi-organ disease affects a number of organs including the lungs which is prone to oxidative stress since it serves as an interface between the body and the host’s external environment, constantly exposed to a number of exogenous oxidants in the air (Thimmulappa et al., 2019). Viral infections including SARS-CoV-2 are associated with increased production of free radicals due to mitochondrial dysfunction as a result of penetration of the virus into host cells.

## The Role of Inflammation in SARS-CoV-2 Pathogenesis

To protect the body against pathogens, cells have the unique ability to identify and restrict the replication of pathogens. The immune response can be broadly divided into the innate immune system and adaptive immune system (Gasteiger and Rudensky, 2014) with innate immune system providing the first line of immune response. Immune response involves a host cells identifying another genome (pathogen) and targeting it for destruction. This is possible because pathogens express unique molecules known as pathogen-associated molecular patterns (PAMPs), which are sensed by host sensors known as pathogen recognition receptors (PRRs) in a very unique mechanism characterized by specificity (Muralidharan and Mandrekar, 2013). Once PAMPs are recognized by PRRs, an array of anti-pathogen immune response is triggered. The series of events involves induction and increased expression of different inflammatory cytokines, chemokines and type I interferons (Kumar et al., 2011).

Toll-like receptors (TLRs) are a prominent class of PRRs, existing as integral glycoproteins with an extracellular luminal ligand binding domain and a cytoplasmic signaling receptor homology domain (Prince et al., 2011). Once a ligand binds to the extracellular domain, receptor oligomerization occurs resulting in a cascade of intracellular signaling events that generates a proinflammatory response to target the pathogen (Prince et al., 2011). TLRs are limited at recognizing intracellular cytosolic pathogens because they are found at cell and organelle surfaces. Thus, cytosolic PRRs exist which carry out TLR-independent recognition of pathogens. For example, the Herpes Simplex Virus (HSV), which is an enveloped DNA virus with HSV-1 and HSV-2 subtypes, is known to cause oral and genital herpes (Govindan, 2014). During HSV infection, HSV virions interact with TLR2 on cell surfaces resulting in increased production of cytokines (Govindan, 2014). During the process of infection, interferons are also activated downstream of TLR9 activity. Viruses have developed mechanisms that alter the effectiveness of PRR-mediated immune responses. Some viruses like HIV and Rabies virus contain NF-kB binding sites in promoter regions which is used to stimulate NF-kB and modulate cellular growth and apoptosis (Kammouni et al., 2012). Thus, the potential role of toll-like receptors in COVID-19 pathogenesis has generated attention as shown from an *in silico* molecular docking study which showed significant binding between the spike protein of SARS-CoV-2 and toll-like receptors especially TLR4 (Choudhury and Mukherjee, 2020).

A limitation of the recognition mechanism of immune response is that pathogens can sometimes escape recognition once they alter an identifying molecular feature like the order of monomers in its polymers (Nathan and Shiloh, 2000). Another limitation of the immune response to parasitic invasion is that significantly increased apoptosis may overcome the expansion and differentiation of lymphocytes, resulting in altered immune responses that enhance parasite survival (Guillermo et al., 2009). These limitations can explain why host cells also use reactive species as a defense mechanism to target pathogens and prevent infection. Increase in plasma pro-inflammatory cytokines such as interleukin 6 (IL-6), interleukin 8 (IL-8) and TNF- α during infectious disease, correlating with increased ROS levels, also suggests that an oxidative mechanism is involved in the immune response (Gasteiger and Rudensky, 2014). Neutrophils, monocytes and macrophages, components of the innate immune response, carry out unique functions necessary for protection against infection. Neutrophils are the most abundant innate immune effector cells which use phagocytosis or oxidizing agents such as NADPH oxidase, to destroy evading pathogens (Muralidharan and Mandrekar, 2013). NADPH oxidase 2 (NOX-2) can generate reactive species, resulting in oxidative stress and thrombotic events in COVID-19 patients (Violi et al., 2020). Elevated levels of cytokines has also been reported in COVID-19 pathogenesis (Liu J. et al., 2020). On entry of SARS-CoV-2 into cells of the respiratory system, viral replication occurs and the innate immune system responds by activating macrophage and dendritic cells which contribute to inflammation. This is evidenced by inflammatory lymphocytic infiltration in lungs of patients on histological examination (Costela-Ruiz et al., 2020). The disease pathogenesis is associated with a ‘cytokine storm’ with elevated levels and activity of interleukin 1β, interleukin 6 and tumor necrosis factor alpha (Cecchini and Cecchini, 2020; Delgado-Roche and Mesta, 2020; Suhail et al., 2020). SARS-CoV-2 pathogenesis has been shown to involve the activation and maturation of IL-1β which then activates IL-6 and TNF-α (Costela-Ruiz et al., 2020).

The immune response is also characterized by assembly and activation of inflammasomes induced by ROS production (Harijith et al., 2014). The activation of inflammasomes causes synthesis of caspase-1, and downstream activation of interleukin 1β and interleukin 18 which are especially implicated in lung injury (Masters, 2013). A prominent member of the inflammasome family is the nucleotide-binding oligomerization domain-like receptor containing pyrin domain 3 (NLRP3) (Abais et al., 2015). Excess ROS generation causes the activation of NLRP3 inflammasome and other cytokines seen in type 2 diabetes and neurodegenerative diseases (Abais et al., 2015). A study on the role of inflammasomes in COVID-19 examined NLRP3 in human peripheral blood mononuclear cells and tissues of postmortem patients (Rodrigues et al., 2020). The results showed active NLRP3 in the samples which was associated with caspase 1, IL-18 activity, disease severity and poor clinical outcomes (Rodrigues et al., 2020), suggesting that NLRP3 could serve as a therapeutic target for COVID-19.

## Therapeutic Potential of Antioxidants in COVID-19 Treatment

**Figure 1:**
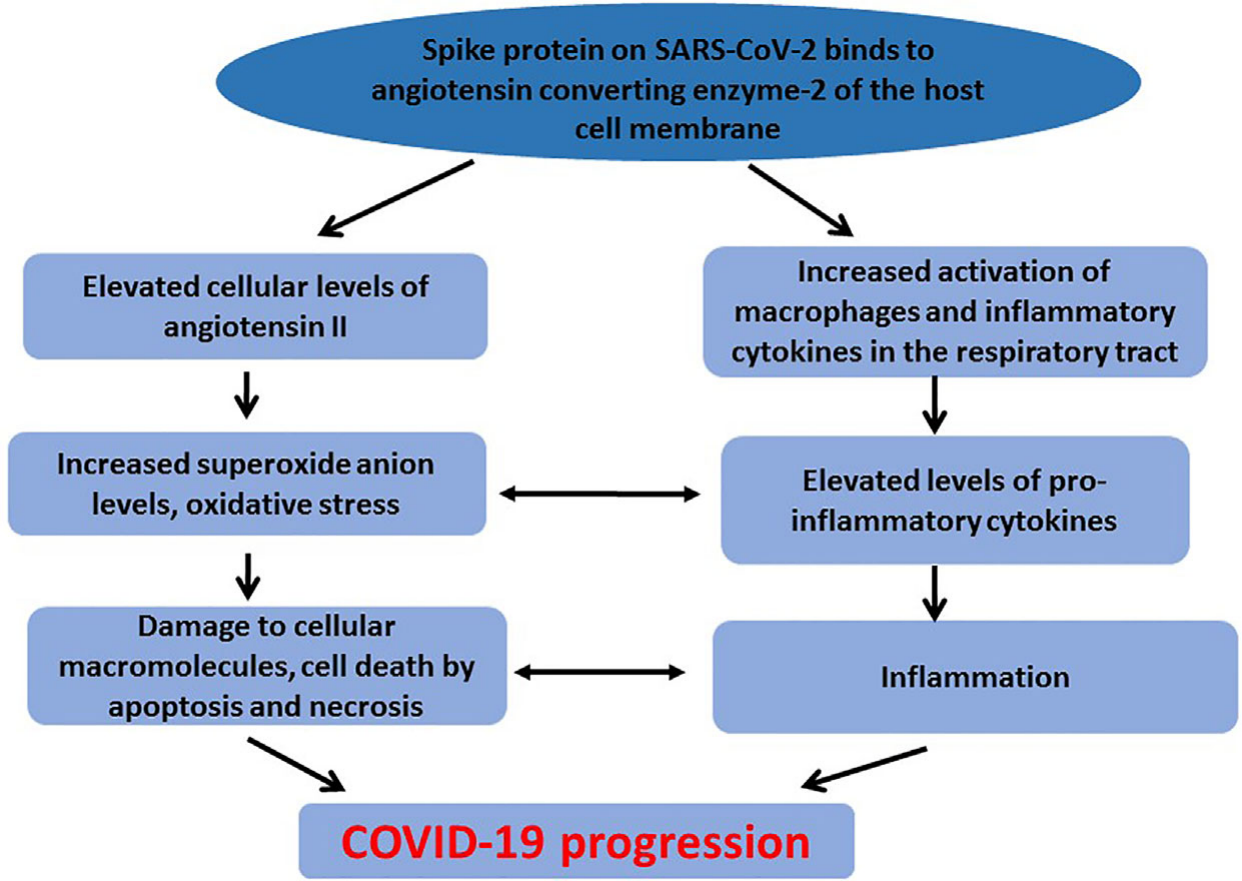
Crosstalk between oxidative stress and inflammation in COVID-19 pathogenesis. To counteract elevated level of ROS, and maintain redox balance, cells are endowed with a number of enzymatic and non-enzymatic antioxidant proteins. These include superoxide dismutase, catalase, glutathione, glutathione peroxidase, thioredoxin, metallothionein among others (Shao et al., 2008). However, when ROS generation exceeds the cellular antioxidant mechanisms, damage to macromolecules still occurs. Underlying diseases associated with oxidative stress and inflammation have been found to complicate COVID-19 progression, suggesting that the compromised redox status of the patients could contribute to the disease progression (Das et al., 2020a). Diabetes is an underlying condition reported to complicate treatment outcomes in COVID-19 patients (Erener, 2020). In diabetic patients infected with SARS-CoV-2, therapeutic strategies that involve ACE or angiotensin receptor inhibitors could aggravate the condition. SARS-CoV-2 is also reported to cause direct damage to pancreatic β-cells, thereby increasing insulin resistance and contributing to severe morbidity and possible mortality of infected diabetic patients (Das et al., 2020b). Hypertension has also been associated with poor clinical outcomes among COVID-19 infected patients (Shibata et al., 2020). Hypertension is associated with left ventricular hypertrophy and fibrosis which could be exacerbated during COVID-19 infection due to oxidative stress and inflammation associated with the disease pathogenesis (Kulkarni et al., 2020). Thus, current therapeutic strategies including the use of ACE-2 inhibitors and ventilators may not produce the expected therapeutic results in this category of patients. Therefore, therapeutic strategies that use natural compounds to counteract oxidative stress and inflammation could be beneficial or could be used as adjuvants (Soy et al., 2020).

The cross talk between oxidative stress and activation of pro-inflammatory cytokines contributes to damage of cellular macromolecules and tissues observed in COVID-19 patients (**Figure 1**). Increased angiotensin II concentration and activity resulting in oxidative stress and damage to cellular macromolecules can activate pro-inflammatory cytokines thereby exacerbating the inflammatory response and contributing to COVID-19 severity (Beltrán-García et al., 2020).

Antioxidants can counteract ROS generation and improve disease treatment through a number of mechanisms. One way is by direct conversion of ROS into inactive forms. Catalase and glutathione peroxidase are enzymes that convert hydrogen peroxide to water and oxygen (Glorieux and Calderon, 2017). Superoxide dismutase converts the active superoxide anion into a relatively less reactive hydrogen peroxide (Sheng et al., 2014). Secondly, antioxidants like glutathione and thioredoxin can donate hydrogen to free radicals, thereby scavenging free radicals (Allen and Bradley, 2011). Thirdly, antioxidants like ferritin and transferrin can reduce ROS generation by chelating transition metals like Fe^2+^ which reacts with molecular oxygen and hydrogen peroxide to generate superoxide anion and hydroxyl radical respectively (Imam et al., 2017). Through these mechanisms, antioxidants can control the levels of ROS during oxidative stress and disease conditions characterized by oxidative stress.

Despite the novel potentials of antioxidants in disease management, studies have shown that antioxidants could be limited in the treatment of certain diseases (Bast and Haenen, 2013). It has even been reported that antioxidant overuse may have little therapeutic effects or could cause detrimental effects (Salehi et al., 2018). These concerns about the efficacy of antioxidant supplementation could be attributed to factors like the varying roles of reactive oxygen species in the pathogenesis of different diseases and the controlled regulation of endogenous antioxidant systems which could reduce systemic response to high doses of dietary antioxidant supplementation (Halliwell, 2013). Reductive stress is the term used to describe a condition in which there is a relative shortage of ROS when compared to reducing equivalents, resulting in deleterious effects due to a breakdown in homeostasis and redox balance (Pérez-Torres et al., 2017). Thus, it has been suggested that at some levels, antioxidants can induce stress in cells when the required homeostatic balance between antioxidants and free radicals in the body is lost (Villanueva and Kross, 2012). It is also known that antioxidant phenols like quercetin during the process of proton donation to free radicals, can form antioxidant radicals which can be stabilized by delocalization of the unpaired electron around the phenol ring to form a stable resonance hybrid (Li Z. et al., 2020). Delocalization of electrons in the attacking radical weakens the new bond and reduces the rate of abstraction, thereby limiting further free radical scavenging ability of the antioxidant radical (Boots et al., 2003).

Studies on antioxidant supplementation reported that *in vitro* administration of epigallocatechin-3-gallate, a component of green tea, in rodent macrophage-like RAW 264.7 and human promyelocytic leukemic HL60 cell lines caused increased generation of hydrogen peroxide, increased oxidative stress and caused genotoxicity due to spontaneous hydrogen peroxide generation of hydrogen peroxide by polyphenols in solution (Elbling et al., 2005). Similar contrasting results on the efficacy of antioxidants in the management of cancer can be seen from a study which reported that dietary carotenoids reduced the risk of lung cancer in male smokers (Holick et al., 2002), while another study reported that administration of oral capsules containing carotenoids increased the incidence of skin cancers in females but not in males (Hercberg et al., 2007). Whether or not these negative effects will be observed in COVID-19 patients is yet to be ascertained.

Despite the challenges and gaps required to be filled on the therapeutic efficacy of antioxidants in the treatment of diseases, a number of antioxidants have been considered for the management of COVID-19. Considering the antioxidant, anti-inflammatory and immunostimulatory properties of vitamin C (ascorbic acid or ascorbate) for instance, the possible therapeutic effects of vitamin C in COVID-19 therapy has received attention (Carr and Rowe, 2020; Hoang et al., 2020). Studies have shown significantly lower vitamin C levels in COVID-19 patients which correlated with disease progression in the patients (Arvinte et al., 2020; Chiscano-Camón et al., 2020). It has also been suggested that the use of vitamin C along with currently used anti-viral and anti-inflammatory drugs could be beneficial to COVID-19 patients (Feyaerts and Luyten, 2020). However, a randomized clinical trial of patients diagnosed with COVID-19 showed no improvement in treatment outcomes when administered vitamin C (Thomas et al., 2021). Yet, an ongoing randomized controlled trial is examining the effects of intravenous high-dose administration of vitamin C in COVID-19 patients on cytokine levels, pulmonary function and hospitalization time (Liu F. et al., 2020).

Vitamin D comprises a number of fat-soluble steroids with anti-inflammatory and immune modulatory properties, for which 1,25-dihydroxyvitamin D is the active form (Xu et al., 2020). The inflammatory and immune suppressive effects associated with COVID-19 pathogenesis has generated research interest into the potential role of vitamin D in COVID-19 therapy (Grant et al., 2020). Serum levels of vitamin D is reportedly depleted in COVID-19 patients and could contribute to severity of disease progression (Arvinte et al., 2020; Radujkovic et al., 2020). Plasma concentrations of 25-hydroxyvitamin D (25(OH)D) in PCR-positive SARS-CoV-2 patients were also reported to be significantly lowered compared to negative patients (D’avolio et al., 2020). A study examined the therapeutic effects of bolus vitamin D supplementation in COVID-19 patients and found that the supplementation correlated with less severe disease progression and enhanced survival rate (Annweiler et al., 2020). It has been shown that Vitamin D can interact with the innate immune system, by activating Toll-like receptors (TLRs) or increasing the levels of cathelicidins and β-defensins, and adaptive immune system, by reducing immunoglobulin secretion by plasma cells and pro-inflammatory cytokines production, thus modulating T cells function (Panfili et al., 2021). Its immune modulating response to the virus might play a role in the prevention and/or treatment to SARS-CoV-2 infection disease in the adult and pediatric population. However, since vitamin D enhances ACE-2 expression, there are concerns that vitamin D supplementation could enhance SARS-CoV-2 binding resulting in aberrant immune response and poor therapeutic outcomes in COVID-19 patients (Cereda et al., 2020). These contrasting predictions require further studies and trials to determine the actual dose of vitamin D and method of administration that could be therapeutic or otherwise. Such studies will also need to consider the stage of the disease and presence or absence of underlying conditions in the study subjects.

The hormone melatonin is a methoxyindole mainly synthesized and secreted by the pineal gland with reported antioxidant, anti-inflammatory and immunomodulatory properties (Cardinali et al., 2020). The hormone functions as a free radical scavenger, protects mitochondria membrane integrity during oxidative stress and counteracts lung injury associated with viral infections *via* interactions with a number of cellular proteins, signaling molecules and enzymes (Juybari et al., 2020). Specifically, melatonin has been shown to block CD147, a glycoprotein implicated in viral invasion mediated lung inflammation (Sehirli et al., 2020). These properties has attracted research interest into the possible protective effects of melatonin as an adjuvant therapy against COVID-19 (Cardinali et al., 2020; Kleszczyński et al., 2020). Currently, a clinical trial is using injectable melatonin formulations in COVID-19 patients to determine the doses and efficacy of melatonin against COVID-19 (Acuña-Castroviejo et al., 2020). Similar studies that take into consideration doses, route of administration and underlying conditions of the study subjects will provide further understanding of the ameliorative potential of melatonin in COVID-19 patients.

N-acetyl cysteine (NAC) is an immediate precursor of glutathione (Poe and Corn, 2020). Glutathione is a tripeptide of cysteine, glutamic acid and glycine with known antioxidant potency (Silvagno et al., 2020). NAC exerts antioxidant effects by directly contributing to the pool of intracellular cysteine and enhancing glutathione synthesis (Poe and Corn, 2020). Based on the ability of glutathione to attenuate oxidative stress, decrease levels of circulating IL-6 and inhibit viral replication reports have suggested that glutathione supplementation could be therapeutic to COVID-19 patients (Guloyan et al., 2020; Silvagno et al., 2020). Similar suggestions have also been made about the possible protective effects that NAC supplementation could have on COVID-19 patients (Poe and Corn, 2020; Rangel-Méndez and Moo-Puc, 2020). However, a double-blind randomized clinical trial with high doses of NAC in COVID-19 patients did not report any significant ameliorative effects in the administered patients when compared to patients administered placebo (de Alencar et al., 2020). Studies are needed to determine if ameliorative effects could be observed in patients administered low doses of NAC.

Curcumin, a hydrophobic polyphenol, is the active constituent of *Curcuma longa* also known as turmeric (Babaei et al., 2020). Curcumin is widely known as a spice and research has shown that it exhibits antioxidant, anti-inflammatory and anti-viral effects (Babaei et al., 2020). *In silico* docking results have shown that the viral spike protein and ACE-2 possess binding affinity to curcumin *via* covalent and non-covalent interactions, suggesting that the compound can interfere with SARS-CoV-2 entry into host cells (Das et al., 2020b). Considering that the ACE-2 receptor is significantly expressed in nasal cells and mucosal cells of the respiratory tract, an emulsion of topically applied curcumin as an adjuvant therapy for COVID-19 has been proposed (Manoharan et al., 2020). An ongoing randomized controlled trial of a capsule containing 500 mg curcumin and 5 mg piperine is being administered to COVID-19 patients to determine possible therapeutic effects on disease severity and inflammatory mediators in the study subjects (Miryan et al., 2020)

Quercetin is a plant flavonoid with reported antioxidant, anti-inflammatory and antiviral effects (Colunga Biancatelli et al., 2020). Although there are research interests on the potential therapeutic effects of quercetin in COVID-19 patients, there is a dearth of information on clinical benefits associated with the supplementation of quercetin in COVID-19 patients (Aucoin et al., 2020; Colunga Biancatelli et al., 2020). Also, resveratrol and polydatin which is the bioavailable form have received attention with respect to COVID-19 therapy due to previously reported antioxidant and anti-inflammatory effects in *in vitro* and *in vivo* models of diseases for which oxidative stress and inflammation are implicated (Bonucci et al., 2020; Lo Muzio et al., 2020). However, in-depth studies are required to establish these suggestions. Studies are also needed to determine the most effective route of administration of antioxidants, taken into consideration the proposed therapeutic mechanism of the antioxidant viz-a-viz specific disease conditions and physiological state of the patients. The highlighted antioxidants with suggested ameliorative potentials for COVID-19 and the knowledge gaps that need to be bridged are summarized in **Table 1**.

**Table 1:** Antioxidants with potential therapeutic effects for COVID-19.

S/No.	Antioxidants	Reported clinical observations in COVID-19 patients	Knowledge gaps	Reference
1	Vitamin C	Significantly low vitamin C levels in serum and plasma	Determining the therapeutic doses of vitamin c to be used in COVID-19 patients at varying levels of oxidative stress	(Arvinte et al., 2020; Chiscano-Camón et al., 2020)
2	Vitamin D	Significantly low vitamin D levels in serum and plasma	Determining the effect of vitamin D supplementation at different doses on ACE-2 expression and response to therapy among COVID-19 patients	(Arvinte et al., 2020; D’avolio et al., 2020)
3	Melatonin	–	Determining levels of melatonin in serum of COVID-19 patients. Correlating exogenous melatonin administration with CD147 activity and response to therapy among COVID-19 patients	(Cardinali et al., 2020; Kleszczyński et al., 2020)
4	N-acetyl cysteine/ Glutathione	A trial study using high doses of NAC reported no significant ameliorative effects	Studies are needed to determine if low doses NAC supplementation can have ameliorative effects in COVID-19 patients	(de Alencar et al., 2020; Poe and Corn, 2020)
5	Curcumin	–	Antioxidant and anti-inflammatory effects of quercetin in COVID-19 patients	(Manoharan et al., 2020; Miryan et al., 2020)
6	Quercetin Resveratrol	–	Anti-inflammatory and immunomodulatory effects of quercetin and resveratrol in COVID-19 patients	(Aucoin et al., 2020; Colunga Biancatelli et al., 2020)

## Conclusion

Oxidative stress and inflammation are interconnected processes that contribute to COVID-19 progression and response to therapy. Natural antioxidants can counteract altered signaling pathways activated during COVID-19 pathogenesis. The highlighted research gaps show that further investigations are needed along this line to provide efficient counteracting strategies to ameliorate severity of the disease and improve treatment outcomes especially in patients with underlying complications.

## Author Contributions

GF - conception and drafting of manuscript. AM - research and drafting of manuscript. OO - research and drafting of manuscript. SM - research and drafting of manuscript. CM - conception and drafting of manuscript. All authors contributed to the article and approved the submitted version.

## Conflict of Interest

The authors declare that the research was conducted in the absence of any commercial or financial relationships that could be construed as a potential conflict of interest.
